# ‘This will bring shame on our nation’: The role of anticipated group-based emotions on collective action

**DOI:** 10.1016/j.jesp.2012.07.011

**Published:** 2013-01

**Authors:** Lee Shepherd, Russell Spears, Antony S.R. Manstead

**Affiliations:** a Department of Psychology, School of Natural Sciences, University of Stirling, Scotland, UK; b School of Psychology, Cardiff University, Wales, UK; c Department of Social Psychology, University of Groningen, The Netherlands

**Keywords:** Anticipated emotion, Collective action, Group-based guilt, Group-based shame, Group-based anger

## Abstract

In three studies we examined whether the anticipation of group-based guilt, shame and anger predicts the desire to undertake collective action against a proposed ingroup transgression. In Studies 1 (N = 179) and 2 (N = 186), the relation between appraising a proposed ingroup transgression as illegitimate and collective action was mediated (or partially mediated) by anticipated group-based shame and anger. In Study 3 (N = 128) participants with high self-investment group identification were less willing to engage in collective action against the prospective ingroup transgression when aversive anticipated group-based emotions were made salient. This effect was mediated by anticipated group-based shame. We discuss the implications of these results with regard to collective action and the morality of intergroup behavior.

## Highlights

► We tested whether anticipated group-based emotions predict collective action. ► Anticipating shame and anger increased protesting against immoral ingroup actions. ► Anticipating group-based guilt did not uniquely predict collective action. ► Anticipated group-based guilt predicted reparations.

## Introduction

In 2002, the then President of the United States (George W. Bush) and the then Prime Minister of Great Britain (Tony Blair) announced that American and British troops were going to be deployed in Iraq to search for weapons of mass destruction and to free the Iraqi people. This announcement led to large-scale protests in Britain and the United States. On 15th February 2003, between 750,000 (police estimate) and 2 million (organizers' estimate) protesters gathered on the streets of London to show their opposition to the war, creating the largest protest in the city's history. On the same day between 100,000 (police estimate) and 375,000 (organizers' estimate) people gathered on the streets of New York to protest against the invasion. Despite these protests by British and American citizens, the invasion of Iraq commenced on 19th March 2003. The current research addresses the question of why people are motivated to act collectively against aversive events that look possible but have not yet happened, and the role of (anticipated) group emotions in this process.

A growing body of research has found that morality is a key aspect of social identity (Ellemers, Pagliaro, Barreto, & Leach, 2008; Leach, Ellemers, & Barreto, 2007; Scheepers, Spears, Manstead, & Doosje, 2009). In this article we develop this work by outlining a self-regulatory process that helps ingroup members to maintain a moral group identity. We argue that aversive anticipated group-based emotions (e.g., shame and anger) promote moral intergroup behavior by signaling the harmful emotional consequences of a proposed ingroup transgression. The desire to avoid these aversive emotions motivates ingroup members to prevent the transgression from occurring. One strategy that may alter the intended behavior of one's group is collective action. We therefore extend the intergroup and collective action literature by arguing that the mere anticipation of an aversive group-based emotion is sufficient to motivate group members (i.e., British and American citizens) to undertake collective action against a proposed ingroup transgression, such as the use of military force against Iran's alleged nuclear weapons program. This hypothesis was tested in three studies.

## The effect of group-based emotions on collective action

People may experience guilt, shame and anger in relation to the actions or attributes of their own group simply through their association with the ingroup, even in the absence of personal responsibility for these actions or attributes (Brown & Cehajic, 2008; Doosje, Branscombe, Spears, & Manstead, 1998; Leach, Iyer, & Pedersen, 2006). These group-based emotions are felt when people appraise their group as responsible for an illegitimate negative act (Branscombe, Doosje, & McGarty, 2002; Iyer, Schmader, & Lickel, 2007; Leach, Snider, & Iyer, 2002). The exact appraisal of the illegitimate action determines which emotion(s) will be evoked.

People are likely to experience group-based guilt when they believe that their group was responsible for an illegitimate action (Branscombe et al., 2002; Doosje et al., 1998). Guilt is regarded as a self-focused emotion because people focus on *their* illegitimate act and the negative affect that *they* are currently experiencing (Harth, Kessler, & Leach, 2008; Iyer, Leach, & Crosby, 2003; Leach et al., 2002; Roseman, Wiest, & Swartz, 1994). Moreover, because guilt is associated with low levels of physical arousal and action readiness (Frijda, Kuipers, & ter Schure, 1989; Roseman et al., 1994) it may also be regarded as a relatively passive emotion (Iyer et al., 2003; Leach et al., 2002, 2006).

Although researchers have debated whether shame maybe derived from actions implying a global (e.g., Niedenthal, Tangney, & Gavanski, 1994; Tangney, 1991; Tracy & Robins, 2006) or specific lapse of identity and reputation (e.g., Gausel & Leach, 2011), there is consensus that, in contrast to guilt, the focus is on the self rather than the behavior (Allpress, Barlow, Brown, & Louis, 2010; Ferguson, Burgman, White, & Eyre, 2007; Lewis, 1971; Tangney, 1992). Shame is therefore likely to be felt when people believe that a transgression tarnishes their image (Ferguson, 2005; Tangney & Dearing, 2002). Similarly, intergroup research has found that group-based shame is likely to be elicited when people believe that an ingroup action tarnishes social identity (Iyer et al., 2007; Lickel, Schmader, Curtis, Scarnier, & Ames, 2005; Lickel, Schmader, & Spanovic, 2007).

Group-based anger is typically felt towards outgroups who have harmed the ingroup (Gordijn, Yzerbyt, Wigboldus, & Dumont, 2006; Van Zomeren, Spears, Fischer, & Leach, 2004; Van Zomeren, Spears, & Leach, 2008; Yzerbyt, Dumont, Wigboldus, & Gordijn, 2003). However, people can also feel angry at their own group when they believe that the ingroup is responsible for harming an outgroup (Iyer et al., 2003; Leach et al., 2006); whereas guilt is a self-focused emotion, ingroup-directed anger is an other-focused emotion that is felt when people are concerned with the effect of the ingroup's actions on the *victimized outgroup*. Moreover, in contrast to guilt, anger is associated with a high level of arousal and action readiness (Frijda et al., 1989; Roseman et al., 1994).

Returning to the Iraq example, some sections of the media suggested that the invasion of Iraq was a ‘front’ for gaining access to the country's oil resources. If the war was thought to be driven by this motivation, the future actions of the ingroup would have likely been appraised as illegitimate. British and American citizens are likely to experience group-based shame when they believe that this action highlights a self-defect that tarnishes their moral identity, guilt when they focus on their group's perceived illegitimate action and its aversive affective consequences, and anger when they believe that the invasion was an avoidable injustice against Iraqi people and focus on the negative consequences of this action for this outgroup.

Social identity theory (Tajfel & Turner, 1979, 1986) postulates that ingroup members are motivated to maintain a positive social identity. All three of the above group-based emotions threaten social identity by associating the ingroup with a transgression (Branscombe, Ellemers, Spears, & Doosje, 1999). However, because a self-defect is more damaging than perceiving a single action to be illegitimate, group-based shame poses a greater threat to social identity than does anger or guilt. In contrast to guilt and anger, group-based shame is likely to be evoked when social identity is tarnished (Iyer et al., 2007; Lickel et al., 2005) and predicts the desire to repair the ingroup's image (Johns, Schmader, & Lickel, 2005; Schmader & Lickel, 2006).

Each of these group-based emotions is associated with a different action tendency. Group-based guilt is associated with the desire to compensate the victimized outgroup (Brown & Cehajic, 2008; Doosje et al., 1998). However, the low action potential and self-reflective nature of this emotion are likely to be insufficient to promote the kinds of effortful behaviors that may resolve the situation, such as collective action (Iyer et al., 2003; Leach et al., 2006). By contrast, the high activation associated with anger motivates group members to undertake collective action in order to resolve the injustice (Livingstone, Spears, Manstead, & Bruder, 2009; Van Zomeren et al., 2004, 2008), even when the transgression was performed by the ingroup (Iyer et al., 2007; Leach et al., 2006).

Shame elicits actions that serve to repair social identity. A traditional account of shame suggests that it promotes self-defensive action tendencies, such as social withdrawal and the externalization of blame (Stuewig, Tangney, Heigel, Harty, & McCloskey, 2010; Tangney, Wagner, Fletcher, & Gramzow, 1992). However, recent research has found that shame can also promote prosocial behaviors that serve to repair one's identity (De Hooge, Breugelmans, & Zeelenberg, 2008; De Hooge, Zeelenberg, & Breugelmans, 2010; Gausel & Leach, 2011; but see Allpress et al., 2010), such as reparations (Brown & Cehajic, 2008; Brown, Gonzalez, Zagefka, Manzi, & Cehajic, 2008). People may also try to repair social identity through collective action. For example, Iyer et al. (2007) found that group-based shame positively predicted British and American people's willingness to undertake collective action supporting a withdrawal of allied forces from Iraq. Although Iyer et al. (2007) suggest that this was a form of distancing, we argue that such action may also reflect the desire to stop a current transgression (e.g., the “Not in Our Name” campaign). Our argument is supported by the fact that Iyer et al. (2007) found that emotions that are not typically associated with social distancing (i.e., group-based anger) also predicted this form of collective action.

Given evidence that British and American people were motivated to protest against the invasion of Iraq when they experience group-based shame and anger (Iyer et al., 2007), it seems reasonable to assume that such group-based emotions may have motivated British and American people to take part in the protests on 15th February. However, the protests took place a month *before* allied forces invaded Iraq, so it seems unlikely that these emotions would have been experienced directly or as a direct result of these events. At the time of the protests the ingroup had not as yet committed any transgressions that might provide the basis for these emotions. We argue that appraising the future behavior of the ingroup as illegitimate might have led people to *anticipate* feeling group-based guilt, shame and anger were their country to invade Iraq, and that the desire to avoid these aversive events and their related emotions motivated people to protest against the invasion. We therefore extend the collective action literature by arguing that the mere anticipation of aversive group-based emotions is sufficient to promote collective action.

It is possible that people directly experienced group-based anger and shame at the fact that their leaders were even considering whether to undertake a transgression. However, in line with Baumeister, Vohs, DeWall, and Zhang (2007) we argue that these directly experienced emotions are likely to promote behavior by signaling the anticipated emotion consequences of this transgression (‘If we are angry that our leaders are even considering this action, we will be furious if they actually undertake it’). We therefore argue that in such circumstances the anticipation of aversive group-based emotions is likely to promote collective action against an impending transgression.

## The effect of anticipated emotions on moral conduct

Despite widespread evidence for ingroup bias, recent research suggests that group members are motivated to see their behavior as moral and to maintain a moral social identity (Ellemers et al., 2008; Leach et al., 2007; Scheepers et al., 2009). At the interpersonal level, Bandura (1986, 1991, 2001) argues that the anticipation of positive and negative self-sanctions serves the self-regulatory function of promoting moral conduct. People are likely to anticipate positive affective rewards (such as self-respect) for undertaking moral actions and negative affective reactions (such as self-contempt) for immoral behavior. The desire to experience positive and avoid negative arousal promotes moral conduct. This approach has been elaborated by subsequent theories (e.g., Baumeister et al., 2007; Damasio, 1994; Haidt, 2001), who argue that the anticipation of moral self-conscious emotions promotes ethical behavior. Moral self-conscious emotions can be defined as emotions that are associated with the evaluation of the morality of the self (Tangney, Stuewig, & Mashek, 2007). People are likely to experience aversive moral self-conscious emotions (such as guilt and shame) when they violate a moral standard (Tangney & Dearing, 2002). Similarly, people may anticipate experiencing these emotions when they believe that a future action would violate a moral standard (Manstead, 2000). The desire to avoid these aversive self-critical emotions motivates people to inhibit immoral behaviors. For example, research has found that anticipated regret, guilt and shame increases condom use (Hynie, MacDonald, & Marques, 2006; Richard, Van der Pligt, & De Vries, 1996; Traeen & Kvalem, 2007) and deters deviant behavior (Grasmick & Bursik, 1990; Rebellon, Piquero, Piquero, & Tibbetts, 2010; Tibbetts, 1997). We extend this literature by stating that people may anticipate *group-based* emotions for future ingroup actions and that the desire to avoid aversive anticipated group-based emotions promotes moral *intergroup* behavior.

### Group-based emotions

Intergroup emotion theory (Smith, 1993) argues that group-based emotions are experienced when people categorize themselves as ingroup members and perceive the intergroup situation in a way that is consistent with an emotional appraisal. As mentioned earlier, categorizing oneself as an ingroup member and appraising the actions of this group as illegitimate is likely to elicit group-based guilt, shame and anger (Branscombe et al., 2002; Iyer et al., 2007; Leach et al., 2006; Lickel et al., 2005). We extend intergroup emotion theory by arguing that *anticipated* group-based guilt, shame and anger are evoked when people categorize themselves as ingroup members and appraise a future ingroup action (e.g., a military intervention against Iran's alleged nuclear weapons program) as illegitimate.

Group-based shame, guilt and anger are aversive, as demonstrated by the numerous ‘identity management strategies’ (Ellemers, Wilke, & Van Knippenberg, 1993; Van Knippenberg, 1989) that people use to avoid them (for an overview, see Branscombe & Miron, 2004). Generally, the intergroup literature has focused on the implementation of these strategies *after* the event has taken place. However, people may also implement identity management strategies (such as legitimization and dehumanization) *before* an event takes place (Bar-Tal, 1990; Staub, 1989). We extend this literature by arguing that people may also implement *pro-social* identity management strategies in an anticipatory fashion. One such strategy involves attempts to stop the transgression from happening in the first place. When people predict (or anticipate) that the future actions of their group would result in them feeling aversive group-based emotions, they should be motivated to try to stop the transgression from occurring. Collective action is one strategy for changing the intended behavior of the ingroup. Thus if people (e.g., British and American citizens) appraise a proposed ingroup action (e.g., the use of military force against Iran's alleged nuclear weapons program) as illegitimate, they may well anticipate feeling group-based guilt, shame and anger arising from this transgression. The anticipation of these aversive group-based emotions may motivate ingroup members to protest against their group's plans (‘Not in Our Name’). The aim of such protest would be to prevent the ingroup from committing a transgression, thereby avoiding the aversive emotions and the threat posed to social identity.

There is a well-established literature on the role of emotions in regulating intergroup behavior (e.g., Amodio, Devine, & Harmon-Jones, 2007; Devine, Plant, & Buswell, 2000; Maitner, Mackie, & Smith, 2006, 2007). We extend this literature by suggesting that *anticipated* group-based emotions also serve a self-regulatory function. In keeping with Baumeister et al. (2007), we suggest that the anticipation of aversive group-based emotions act to deter immoral intergroup behavior.

Some anticipated group-based emotions should be stronger predictors of collective action than others. We argue that the desire to avoid the social identity threat signaled by anticipated group-based shame and the high level of agitated arousal that is anger should motivate group members to undertake collective action against a proposed ingroup transgression. By contrast, the relatively low level of agitation that is anticipated group-based guilt may be insufficient to motivate people to undertake effortful action tendencies (such as collective action) and may instead promote less effortful action tendencies, such as reparation (Iyer et al., 2003; Leach et al., 2006). We tested these hypotheses in Study 1.

## Study 1

The aim of Study 1 was to assess whether appraising a future ingroup action as illegitimate would elicit anticipated group-based guilt, shame and anger, and whether these emotions would promote collective action against the impending transgression. Participants were informed that the ingroup (the English) was planning to charge students from an outgroup (the Welsh) additional tuition fees for studying at an English university. The future-oriented nature of an illegitimate action in this situation had the potential to elicit the anticipated group-based emotions. We hypothesized that illegitimacy would positively predict anticipated group-based guilt, shame and anger, and that the latter two emotions would, in turn, predict collective action against the proposed ingroup transgression.

### Method

#### Participants

A total of 179 undergraduate and postgraduate students from Cardiff University (29 men and 150 women) participated in this research in exchange for course credit or £1 (approximately $1.60). Their ages ranged from 18 to 38 years, with a mean age of 20.15. All participants were English nationals.

#### Materials

##### Illegitimacy appraisal

Three items were used to assess illegitimacy: ‘It would be wrong for the English to charge extra tuition fees to the Welsh,’ ‘It would be immoral for the English to act in this way,’ and ‘It would be morally unacceptable for the English to treat the Welsh like this’ (*α* = .91). These items were rated on a 7-point Likert scale (1 = *strongly disagree*, 7 = *strongly agree*).

##### Anticipated group-based emotions

Three anticipated group-based emotions were measured: guilt, shame and anger. The guilt and shame items were adapted from Lickel et al. (2005). The guilt items were ‘guilty,’ ‘regret,’ and ‘remorse’ (*α* = .85). The shame items were ‘ashamed,’ ‘uncomfortable,’ and ‘embarrassed’ (*α* = .89). Anger was assessed using the following items: ‘angry,’ ‘annoyed,’ and ‘outraged’ (*α* = .92). Participants were asked: ‘If the English were to charge the Welsh an extra £1250 tuition fees for studying in England, to what extent would you feel [emotion word]?’ Participants responded to these items on 7-point scales (0 = *not at all*, 6 = *extremely*).

##### Collective action intentions

We adapted three items from Van Zomeren et al. (2004) to assess the participant's intentions to perform collective action: ‘I would like to participate in a demonstration against this proposal,’ ‘I would like to participate in raising our collective voice to stop this proposal,’ and ‘I would like to do something with other English people to stop this proposal’ (*α* = .93). Participants rated the extent to which they endorsed these items on a 7-point scale (1 = *not at all*, 7 = *extremely*).

#### Procedure

The study was completed online. After consent had been given, participants read a report stating that universities were suffering from a lack of funding, which could have serious consequences for the standard of teaching. One solution that was being considered by the UK government (in England) was to raise tuition fees for non-English students studying at English universities. However, this would mean that if non-English UK students, such as the Welsh, were to study in England they would be expected to pay more for their education than their English counterparts. To ensure that this discrimination was perceived as illegitimate, participants were informed that the Welsh Government was *not* planning to introduce proposals to increase tuition fees paid by English students studying in Wales. This information was followed by a series of multiple-choice comprehension questions. Participants then completed the illegitimacy, anticipated group-based emotion and collective action measures.

### Results

Nine participants answered more than half of the comprehension questions incorrectly. Removing these participants from the data did not alter the results in any substantive way, so their data were retained. A logarithmic transformation was performed on the illegitimacy variable prior to further analysis to correct for moderate negative skew.

#### Confirmatory factor analysis

We conducted a confirmatory factor analysis to determine the construct validity of the anticipated group-based emotions and hypothesized that anticipated group-based shame, guilt and anger would be three distinct constructs. This three-factor solution was contrasted with a single-factor solution and three two-factor solutions. The three-factor solution provided a significantly better fit with the data than these alternative solutions (see Table 1), confirming that anticipated guilt, shame and anger were indeed separate constructs.

#### Multicollinearity

Because the correlations between the three negative anticipated emotions were high (see Table 2), we calculated the tolerance and variance inflation factors (VIF) to determine whether our dataset was biased by multicollinearity (i.e. tolerance values less than .20; Cohen, Cohen, West, & Aiken, 2003; Menard, 1995). Similarly, VIF values greater than 10 suggest a multicollinearity issue (Myers, 1990). When the illegitimacy and anticipated emotions were entered into a single regression analysis the tolerance values ranged from .25 to .66 and the VIF values ranged from 1.52 to 3.93, suggesting that the dataset was not affected by multicollinearity.

#### Structural equation modeling

##### Hypothesized model

Based on the above rationale, we hypothesized that appraising the situation as illegitimate would positively predict guilt, shame and anger, and that the latter two emotions would positively predict collective action. This model was assessed using AMOS 19 software (Arbuckle, 2010). The model tests were based on the covariance matrix and maximum likelihood estimation was used. The chi-squared value was non-significant, *χ*^2^(1, N = 179) = 2.07, *p* = .150, suggesting that the fit between the data and the model was good. This was confirmed by the other fit indices: goodness-of-fit index (GFI) = 1.00, adjusted goodness-of-fit index (AGFI) = .93, comparative fit index (CFI) = 1.00, normed fit index (NFI) = 1.00, and root-mean-square error of approximation (RMSEA) = .078.^1^

**Fig. 1:**
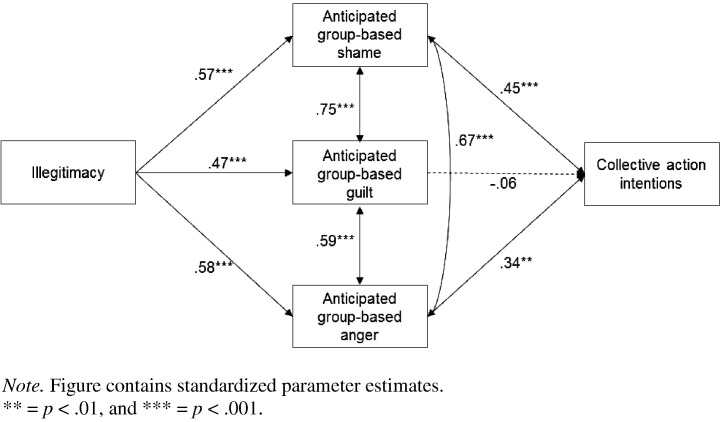
Model for the pathways to collective action against a proposed ingroup transgression (Study 1). **p < .01, and ***p < .001.

As shown in Fig. 1, illegitimacy positively predicted each of the anticipated group-based emotions. Anticipated group-based shame and anger, in turn, positively predicted intentions to perform collective action. The relationship between anticipated group-based guilt and collective action was not significant, implying that this emotion did not uniquely predict protesting. Further analysis revealed that in the fully saturated model anticipated group-based shame and anger predicted collective action, *β* = .42, *p* < .001 for shame and *β* = .30, *p* < .001 for anger, but that illegitimacy and anticipated group-based guilt were non-significant predictors, *β* = − .05, *p* = .573 for guilt and *β* = .10, *p* = .149 for illegitimacy. The fact that the bivariate relationship between illegitimacy and collective action was significant but the direct pathway in the saturated model was non-significant suggests that anticipated group-based shame and anger fully mediated the relationship between illegitimacy and collective action. Moreover, we tested the significance of the indirect pathways of illegitimacy to collective action via the anticipated emotions using 95% bias-corrected confidence intervals, calculated using 5000 bootstrap resamples (see Preacher & Hayes, 2004, 2008). This analysis was conducted in AMOS.^2^ There was a significant indirect pathway through anticipated group-based shame (CI_95_ = .01, .14, *p* = .010) and anger (CI_95_ = .02, .15, *p* = .002). However, the indirect pathway through anticipated group-based guilt was non-significant (CI_95_ = − .01, .03, *p* = .588). The results reflect the fact that anticipated group-based shame and anger (but not guilt) mediated the relationship between illegitimacy and collective action.

##### Alternative models

An alternative model assessed whether the anticipated group-based emotions predicted collective action via the illegitimacy appraisal. We replaced the direct pathways between the anticipated group-based emotions and collective action with an indirect pathway through illegitimacy. The fit indices suggested that this model did not fit the data well: *χ*^2^(3, N = 179) = 74.55, *p* < .001, GFI = .88, AGFI = .40, CFI = .87, NFI = .87, and RMSEA = .366. The model fit for the hypothesized model was significantly better than this alternative model, *χ*^2^(2, N = 179) = 72.48, *p* < .001. The Akaike's information criterion (AIC) was lower for the hypothesized model (30.07) than for the alternative model (98.55), suggesting that the original model was superior.

A second alternative model assessed whether the relationship between the illegitimacy appraisal and anticipated emotions was mediated by intentions to undertake collective action against a proposed ingroup transgression. This model also did not fit the data well: *χ*^2^(3, N = 179) = 36.64, *p* < .001, GFI = .93, AGFI = .65, CFI = .94, NFI = .94, and RMSEA = .251. The hypothesized model fitted the data significantly better than this alternative model, *χ*^2^(2, N = 179) = 34.57, *p* < .001. The AIC for this model (60.64) was lower than that of the hypothesized model, suggesting that the latter was superior.

### Discussion

In Study 1 we found that anticipated group-based guilt, shame and anger were distinguishable constructs and that appraising a future ingroup action as illegitimate positively predicted these three anticipated emotions. Moreover, anticipated group-based shame and anger (but not guilt) positively predicted collective action against a proposed ingroup transgression and mediated the relationship between illegitimacy and collective action. These results extend the intergroup literature by showing that anticipated group-based guilt, shame and anger are experienced when people appraise a future ingroup action as illegitimate, and that the anticipation of these aversive group-based emotions is sufficient to promote collective action against such actions.

A possible limitation of this study is that the participants were English students studying in Wales. The close proximity between the participants and the victimized outgroup may have increased the likelihood of the anticipated group-based emotions being evoked and strengthened their relationship with collective action. A second limitation is that we did not measure action tendencies associated with anticipated group-based guilt. In line with previous research on directly experienced emotions (Iyer et al., 2007; Leach et al., 2006), we argue that the low action potential and self-focused nature of anticipated group-based guilt is more likely to promote less effortful action tendencies (such as reparation) than collective action. This can be interpreted as a ‘minimum effort’ defensive strategy, designed to alleviate any guilt that may be experienced for the future actions of one's group. The aim of Study 2 was to address these limitations and to replicate the findings of Study 1.

## Study 2

There were two differences between Studies 1 and 2. First, we altered the intergroup context. At the time of planning Study 2, the United Nations (UN) was debating how best to deal with Iran's alleged nuclear weapons program. The then UK Foreign Secretary (David Miliband) stated that he would not rule out the use of military force against Iran if they did not start complying with UN sanctions. We used this context because of the parallels with the invasion of Iraq and because of the distance between the perpetrating ingroup and victimized outgroup. Second, we measured people's willingness to compensate the victimized outgroup for any negative effects of the ingroup's actions.^3^ In line with Study 1, we hypothesized that anticipated group-based shame and anger (but not guilt) would positively predict collective action against an impending ingroup transgression. Moreover, based on previous research (Brown & Cehajic, 2008; Brown et al., 2008; Iyer et al., 2007), we hypothesized that all three anticipated group-based emotions would predict reparation intentions.

### Method

#### Participants

A total of 186 participants (138 males and 48 females) completed the online study. Participants were recruited through an advertisement on a social networking website (Facebook). The age ranged from 18 to 64 years, with a mean age of 24.05. All participants were British nationals.

#### Materials

##### Illegitimacy

Participants were asked to rate the extent of their agreement with each of the following items: ‘It would be wrong/unjust/legitimate (reversed)/morally acceptable (reversed) for the British to bomb Iran's nuclear facilities’ (*α* = .93). These items were rated on a 7-point Likert scale (1 = *strongly disagree*, 7 = *strongly agree*).

##### Anticipated group-based emotions

The anticipated group-based shame, guilt and anger terms were identical to those used in Study 1. All three scales were reliable (*α*s = .91 for shame; .90 for guilt, and .96 for anger). Participants were asked: ‘If the British were to bomb Iran's nuclear facilities to what extent would you feel [emotion word]?’ All the items were rated on a 7-point scale (0 = *not at all*, 6 = *extremely*).

##### Collective action intentions

Intentions to engage in collective action were assessed using three items: ‘I would like to participate in a demonstration against Britain bombing Iran's nuclear facilities,’ ‘I would like to participate in raising our collective voice to prevent Britain from bombing Iran's nuclear facilities,’ and ‘I would like to do something with other British people to show our opposition to Britain bombing Iran's nuclear facilities’ (*α* = .96). These items were rated on a 7-point scale (1 = *not at all*, 7 = *very much*).

##### Reparation intentions

The desire to compensate Iranian people for any negative consequences that the bombings might have was assessed using the following 4 items (*α* = .83): ‘If Britain were to bomb Iran's nuclear sites, resulting in civilian casualties, how willing would you be to send aid to the victims/donate money to Iranian humanitarian aid charities/sign a “book of apology”/support a government proposal to send aid to the victims?’ All items were rated on a 7-point scale, ranging from 1 (*not at all*) to 7 (*extremely*).

#### Procedure

At the beginning of the session, participants were informed that the study concerned their thoughts about the current situation in Iran. Participants read a brief report summarizing Iran's alleged nuclear missile program. This outlined the allegation that Iran was developing nuclear weapons, and described the sanctions imposed on Iran by the UN, together with Britain's stance on this issue. The report said that the UK Foreign Secretary stated that he would not rule out the use of military force against Iran. To make this more concrete, participants were told that British forces might bomb Iran's nuclear facilities if they did not start to comply with the UN. This information was followed by a series of comprehension questions. The illegitimacy, anticipated group-based emotion, collective action, and reparation measures were then completed.

### Results

To correct for skew, we performed a logarithmic transformation on the reparation variable prior to further analysis. The three anticipated group-based emotions were highly correlated (see Table 3). A tolerance value of .14 indicated that including all three emotions in a single regression model would create multicollinearity (Cohen et al., 2003; Menard, 1995). To avoid multicollinearity, we analyzed the three anticipated group-based emotions in separate models.

#### Structural equation modeling

**Fig. 2:**
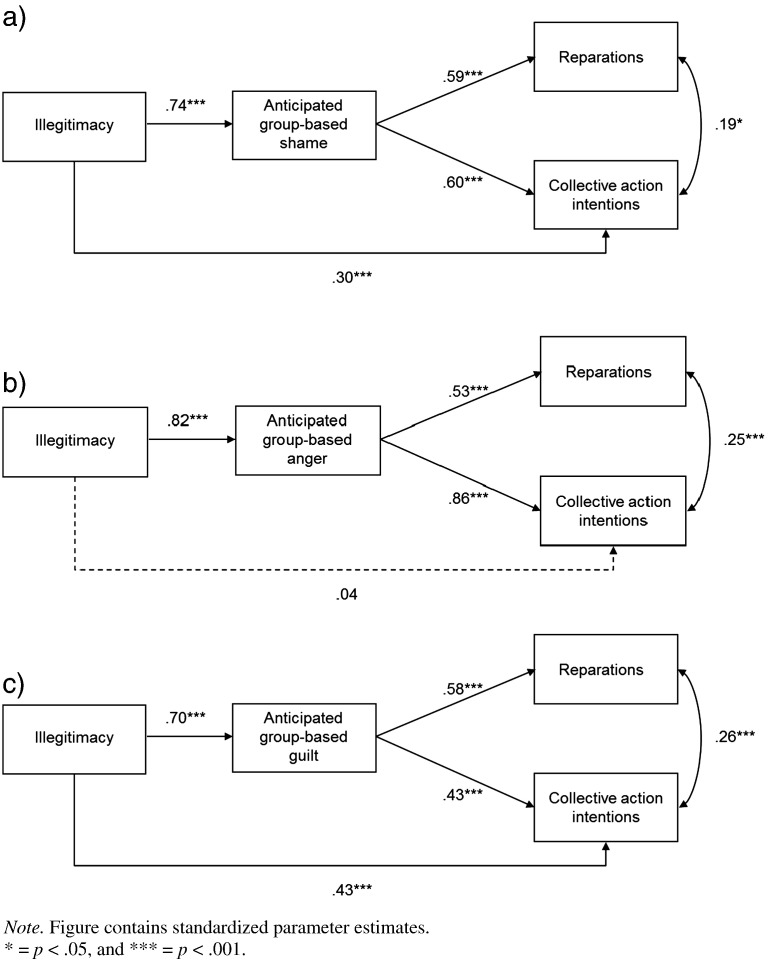
Model for the pathways to collective action against a proposed ingroup transgression and reparations (Study 2). *Note*. Figure contains standardized parameter estimates. *p < .05, and ***p < .001.

We hypothesized that appraising a proposed ingroup transgression as illegitimate would elicit anticipated group-based guilt, shame and anger, and that shame and anger (but not guilt) would predict collective action. We also hypothesized that all three anticipated group-based emotions would predict reparation intentions. This model was assessed using AMOS 19 software (Arbuckle, 2010). The model tests were based on the covariance matrix and maximum likelihood estimation was used. As noted above, the anticipated group-based emotions were analyzed in three separate models (see Fig. 2).^4^

##### Anticipated group-based shame

For the anticipated shame model (Fig. 2a), the chi-squared value was non-significant, *χ*^2^(1, N = 186) < 0.01, *p* = .988, suggesting that the fit between the data and the model was good. This was confirmed by other fit indices: comparative fit index (CFI) = 1.00, normed fit index (NFI) = 1.00, and root-mean-square error of approximation (RMSEA) < .001. Illegitimacy positively predicted anticipated group-based shame, which positively predicted collective action and reparations. The indirect pathways from illegitimacy to collective action and reparation via shame were assessed using 95% bias-corrected confidence intervals, calculated using 5000 bootstrap resamples. The confidence intervals for the indirect pathways to collective action and reparation did not include zero (CI_95_ = .30, .59 and CI_95_ = .34, .53, respectively), indicating that both indirect pathways were significant (*p* < .001 for collective action, and *p* < .001 for reparation). These results suggest that anticipated group-based shame mediates the relationship of illegitimacy to collective action and reparation either partially or fully, respectively.

##### Anticipated group-based anger

The anticipated anger model (Fig. 2b) fitted the data well: *χ*^2^(1, N = 186) = 0.01, *p* = .930, CFI = 1.00, NFI = 1.00, and RMSEA < .001. Illegitimacy positively predicted anticipated group-based anger, which, in turn, predicted collective action and reparation. Moreover, in contrast to the other anticipated emotions, the direct pathway from illegitimacy to collective action was non-significant. The 95% bias-corrected confidence intervals revealed that both indirect pathways from illegitimacy to collective action and reparation (via anger) were significant (CI_95_ = .61, .80, *p* < .001, and CI_95_ = .33, .53, *p* < .001, respectively). This suggests that anticipated group-based anger mediates the relationship of illegitimacy to collective action *and* reparation.

##### Anticipated group-based guilt

The anticipated group-based guilt model (Fig. 2c) fitted the data well: *χ*^2^(1, N = 186) = 0.50, *p* = .478, CFI = 1.00, NFI = 1.00, and RMSEA < .001. Illegitimacy predicted anticipated group-based guilt, which, in turn, predicted collective action and reparation.^5^ The 95% bias-corrected confidence intervals revealed that the indirect pathways from illegitimacy to collective action and reparation (via guilt) were significant (CI_95_ = .19, .43, *p* < .001, and CI_95_ = .31, .49, *p* < .001, respectively). However, the indirect pathway from illegitimacy to collective action via guilt should be interpreted with caution because this indirect effect was non-significant when shame or anger were entered into the model (see Footnote 5).

#### Alternative model

Because of the high correlations between the anticipated emotions, the alternative model assessed whether the relationship between illegitimacy and collective action was mediated by a latent generalized negative affect variable. It should be noted that this model had additional variables to those outlined above and could therefore not be contrasted with these models. However, we assessed this alternative model to determine how well it fitted the data. In this model the three anticipated emotions were observed indicators of a latent generalized negative affect variable. All other pathways were identical to the hypothesized model. The chi-squared value was significant, *χ*^2^(7, N = 186) = 46.80, *p* < .001, suggesting that the model did not fit the data well. This was confirmed by the other fit indices. The CFI (.96) and NFI (.95) were adequate, but the RMSEA value (.175) implied that the model did not fit the data well.

### Discussion

In line with the results of Study 1, we found that illegitimacy significantly predicted all three anticipated emotions, and that anticipated group-based shame and anger positively predicted collective action against a proposed ingroup transgression. Moreover, the relation between illegitimacy and collective action was mediated by anticipated group-based anger and partially mediated by anticipated group-based shame. Contrary to Study 1, we found that anticipated group-based guilt predicted collective action, and that the relationship between illegitimacy and collective action was partially mediated by anticipated group-based guilt. This was most likely due to the shared variance between guilt, shame and anger. Indeed, the relationship between guilt and collective action and the indirect pathway via guilt became non-significant once shame or anger was entered into this model (see Footnote 5), consistent with the findings of Study 1.We also found that all three anticipated group-based emotions positively predicted reparation intentions.

A technical problem in the present study was the multicollinearity. Further analysis revealed that this was due to a high correlation between anticipated group-based shame and anger (see Footnote 5). Associations between shame and anger are well documented in the interpersonal (Lewis, 1971; Scheff, 1988; Stuewig et al., 2010) and intergroup (Iyer et al., 2007) literature. In the present research we believe that it reflected the fact that group members anticipated feeling angry if the transgression were to be undertaken because it posed a threat to social identity. A question that needs to be addressed is why this multicollinearity was not evident in Study 1. In Study 2 we recruited participants through an advertisement on a social networking site. Unlike the undergraduate students in Study 1, these people are likely to sign up to the study based on their interest in the issue under investigation. These participants were likely to have a strong opinion about Iran, resulting in more polarized responses. This polarized response may have reduced variability in the dataset, increasing the likelihood of multicollinearity. This may also explain why our attempt to manipulate (il)legitimacy was not successful (see Footnote 3): those signing up were already likely to have established views on the legitimacy or otherwise of any intervention in Iran.

The results of Studies 1 and 2 provide support for the idea that anticipated group-based emotions promote collective action. A logical next step is to determine the factors that influence anticipated group-based emotions. People's willingness to accept negative information about their group is dependent on their level of ingroup identification (Branscombe et al., 1999; Doosje, Branscombe, Spears, & Manstead, 2006; Doosje et al., 1998). Leach et al. (2008) suggest that identification consists of two superordinate components: *self-investment*, defined as the value and emotional significance attached to the ingroup, as well as the importance and salience of this membership; and *self-definition*, defined as the perception of commonality and similarity within the ingroup and between group members. In positive circumstances, high self-investment in a group is rewarding. However, in negative circumstances greater investment in a group results in more negative affective consequences. Because of this, people with high self-investment are typically motivated to reaffirm a positive ingroup identity and are likely to legitimize negative ingroup actions in order to protect their group and avoid aversive group-based emotions (Doosje et al., 1998; Leach et al., 2008; Leidner, Castano, Zaiser, & Giner-Sorolla, 2010; Roccas, Klar, & Liviatan, 2006). Unlike self-investment, self-definition is not directly related to the desire to maintain a positive ingroup identity.

As stated earlier, the anticipation of negative group-based emotions signals that a proposed ingroup action poses a threat to social identity. People with high self-investment are likely to justify an ingroup transgression in order to protect social identity. As a result, such people should anticipate negative group-based emotions to a lesser extent and thus be less willing to engage in collective action against the transgression. People with low self-investment, on the other hand, are less motivated to protect social identity by justifying a proposed ingroup transgression and are more likely to implement prosocial strategies to protect their small (but significant) investment in the ingroup (Doosje et al., 1998, 2006). By contrast, people with high self-definition are unlikely to justify the ingroup's actions because this component of identification is not directly related to the desire to maintain a positive social identity. These hypotheses were tested in Study 3.

A possible limitation of Studies 1 and 2 is that we focused on negative anticipated emotions. It may be possible that anticipating *positive* emotions for the proposed ingroup transgression would inhibit collective action against the transgression. Group members may experience positive emotions in relation to negative actions directed towards an outgroup when this group is believed to pose a threat to the ingroup (Branscombe & Wann, 1994; Leach et al., 2002). In such circumstances people are likely to justify negative actions in order to eradicate the potential threat. This justification may result in ingroup members anticipating positive emotions for undertaking future transgressions (Maitner et al., 2007). For example, the allegation that Iran possesses nuclear weapons could be seen as a threat to the Western world. Ingroup members may justify military intervention by suggesting that Iran threatened world peace. The use of military force, therefore, becomes justified because it serves a moral function. This might lead some people to anticipate positive group-based emotions for undertaking this strategy, making people less likely to protests against such actions.

## Study 3

Study 3 differed from Study 2 in three respects. First, we measured ingroup identification using the Leach et al. (2008) measure. Second, we investigated the role of positive anticipated group-based emotions on collective action. Three positive emotions were measured in this study: pride, relief and feeling emboldened. The positive arousal that is feeling emboldened arises when people believe that they are superior to and have control over another party. This emotion may be more relevant than pride in the present context because it is related to ingroup superiority. Third, we directly manipulated the anticipated group-based emotions. Previous research has manipulated anticipated emotions using the ‘mere measurement effect’ (O'Carroll, Dryden, Hamilton-Barclay, & Ferguson, 2011; O'Carroll, Foster, McGeechan, Sandford, & Ferguson, 2011; Richard et al., 1996; Sandberg & Conner, 2009). The idea is that simply asking people to state the extent to which they anticipate a specific emotion for undertaking an action increases the salience of this anticipated emotion in the decision making process and the likelihood of it affecting subsequent behavior. We refer to this as a salience manipulation. In the emotion salient condition, participants rated the extent to which they would feel the anticipated group-based emotions following the proposed ingroup transgression *before* stating their willingness to engage in collective action. In the control condition, the anticipated emotions were rated *after* participants stated their willingness to engage in collective action. In the emotion salient condition the anticipated emotions should be more prominent when deciding whether to undertake collective action against the impeding transgression and have a greater effect on subsequent behavior than in the control condition.

We also manipulated the valence of the anticipated group-based emotions by asking participants to rate the extent to which they anticipated *either* positive *or* negative group-based emotions for the ingroup transgression. Positive anticipated group-based emotions signal that an action is likely to result in positive emotions. Therefore, when participants rate the extent to which they anticipate positive anticipated group-based emotions for the use of military force against Iran, they are likely to incorporate the positive emotional consequences of this action into the decision about whether or not to protest against the invasion. The desire to experience these positive consequences is likely to stop group members from protesting against such action, because such a protest might reduce the likelihood of the invasion occurring, along with its positive emotional consequences. Increasing the salience of positive anticipated group-based emotions should therefore decrease the likelihood of group members undertaking collective action against the invasion of Iran. Both people with high and low self-investment are presumably motivated to experience these positive emotions. As a result, the reduction in collective action should not be dependent on self-investment.

The effect of increasing the salience of negative anticipated group-based emotions on collective action is likely to be more complex because this effect is likely to be dependent on the ingroup member's level of self-investment. People with high self-investment are likely to legitimize ingroup transgressions in order to protect social identity (Leach et al., 2008). Therefore, when negative anticipated group-based emotions are salient, people with high (but not low) self-investment should be inclined to justify the ingroup transgression in order to avoid social identity threats and these self-critical emotions. This justification is likely to inhibit anticipated group-based guilt, shame and anger, thus creating a negative relationship between self-investment and the aversive anticipated emotions. Studies 1 and 2 demonstrate that anticipated group-based shame and anger promote collective action. By justifying the transgression, people with high self-investment are less likely to anticipate these emotions, thereby reducing the likelihood that they will undertake collective action against this transgression.

Previous research on self-critical group-based emotions has shown that people with low identification are more likely than high identifiers to undertake action (e.g., Doosje et al., 1998, 2006). Although people with low self-investment have less riding on the valence of the ingroup's identity, they are still motivated to protect their small (but significant) investment in the ingroup, or to atone for, rather than justify its negative actions. The main difference between people with low and high self-investment is thus that low investors are more likely to implement strategies such as collective action and less likely to justify ingroup behavior (Leach et al., 2008). The salience of the negative anticipated emotions is therefore unlikely to reduce the extent of collective action undertaken by people with low self-investment. Because people with high (but not low) self-investment are likely to justify the transgression, there should be a negative relationship between self-investment and collective action when the negative anticipated emotions are salient. When the negative anticipated group-based emotions are not salient, the proposed transgression is less threatening, because negative anticipated group-based emotions have not signaled the aversive emotional consequences of military action and the social identity threat that it poses. People with high self-investment should therefore be less likely to justify the transgression. As a result, it should not be the case that self-investment negatively predicts the aversive anticipated emotions and collective action. Furthermore, because self-definition is unrelated to the desire to maintain a positive social identity, we hypothesized that it would not moderate the effects of the salience and valence manipulations.

### Method

#### Participants and design

A total of 128 undergraduate students (11 males and 117 females) participated in this study in exchange for course credit. Participants' ages ranged from 18 to 33 years, with a mean age of 19.47. A 2 (salience: salient vs. control) × 2 (valence: positive vs. negative) × continuous moderating variable (self-investment or self-definition) between-subjects factorial design was used. In the positive valence condition participants rated the extent to which they anticipated feeling positive group-based emotions (pride, feeling emboldened, and relief) if Britain were to bomb Iran's nuclear facilities. In the negative valence condition participants rated the extent to which they anticipated negative group-based emotions (guilt, shame, and anger) if this were to happen. In the salient condition the anticipated emotion ratings were measured before collective action intentions. In the control condition the anticipated emotions were measured after collective action. Participants were randomly assigned to conditions. The dependent variable was the participant's willingness to engage in collective action.

#### Materials

##### Ingroup identification

In the Leach et al. (2008) 14-item scale, self-investment is assessed using 10 items, such as ‘Being British is an important part of how I see myself’ and ‘I am glad to be British’ (*α* = .93); and self-definition is assessed using 4 items (e.g. ‘I am similar to the average British person,’ and ‘British people have a lot in common with each other;’ *α* = .85). All items were rated on a 7-point Likert scale (1 = *strongly disagree*, 7 = *strongly agree*).

##### Anticipated group-based emotions, collective action and reparation

A new item was added to each of the negative emotion scales, to increase the strength of the manipulation. The additional guilt, shame and anger items were ‘sorry,’ ‘humiliated,’ and ‘furious,’ respectively. All three scales were reliable (*α*s = .87 for guilt; .87 for shame, and .88 for anger). The pride items were ‘proud,’ ‘triumphant,’ ‘victorious,’ and ‘jubilant’ (*α* = .89). The relief items were ‘relaxed,’ ‘relieved,’ ‘reassured,’ and ‘secure’ (*α* = .89). Feeling emboldened was assessed by ‘bold,’ ‘superior,’ ‘fearless,’ and ‘powerful’ (*α* = .83). Participants were asked: ‘If the British were to bomb Iran's nuclear facilities to what extent would you feel [emotion word]?’ These items were assessed on a 7-point scale (0 = *not at all*, 6 = *extremely intensely*). The collective action and reparation measures were identical to those used in Study 2.

#### Procedure

After consent had been given, participants completed the identification scale. This was followed by the information about Iran's alleged nuclear missile program. This described Iran's defiance of UN sanctions and their alleged tests of nuclear weapons. It concluded by stating that the British government has said that it would not rule out the use of military force against Iran if it did not start to comply with UN sanctions. Participants were informed that Britain *might* bomb Iran's nuclear facilities if they did not start to cooperate with the UN. A series of comprehension questions were then completed to ensure that the participant had understood this information.

In the salient conditions the anticipated group-based emotion measure was then completed. In the positive emotion salient condition the positive emotion scales were completed. In the negative emotion salient condition the negative emotion scales were completed. The collective action and reparation intention scales were then completed. In the control conditions these latter scales were rated before the anticipated emotion measures. When all measures had been completed participants were thanked and debriefed.

### Results

Removing three participants who answered more than half the comprehension questions incorrectly did not substantially alter the results, so their data were retained. To correct for positive skew, a square-root, reciprocal, and logarithmic transformation was performed on the relief, pride, and feeling emboldened variables, respectively, prior to further analysis. To correct for negative skew, a square-root transformation was performed on the self-investment variable before further analysis. Two 2 (salience: salient vs. control) × 2 (valence: negative vs. positive) × continuous identification measure (self-investment *or* self-definition, centered) ANOVAs were performed on collective action, one for each identification measure.

#### Self-investment

##### Collective action

**Fig. 3:**
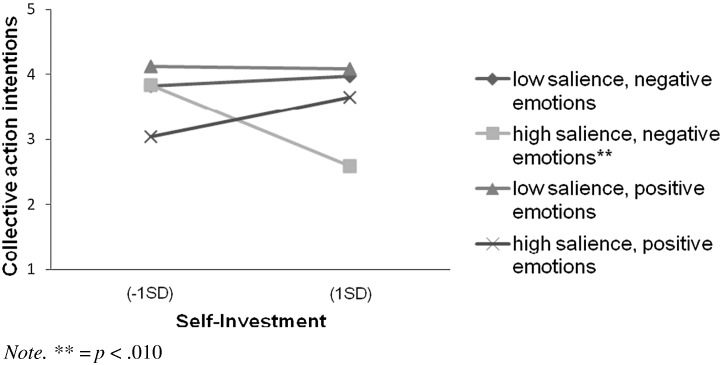
Interaction of salience, valence and self-investment on collective action (Study 3). Error bars = ± 1*SE*. *Note*. **p < .010.

There was a significant main effect of salience, *F*(1, 120) = 9.81, *p* = .002, *η*_p_^2^ = .08. The main effect of valence was not significant, *F*(1, 120) = 0.72, *p* = .399, *η*_p_^2^ = .01. Self-investment did not predict collective action, *F*(1, 120) = 1.10, *p* = .296, *η*_p_^2^ = .01. The valence by self-investment interaction was marginally significant, *F*(1, 120) = 3.44, *p* = .066, *η*_p_^2^ = .03. These results were qualified by a significant three-way interaction between salience, valence and self-investment, *F*(1, 120) = 4.36, *p* = .039, *η*_p_^2^ = .04.^6^ Simple slopes analysis revealed that this significant interaction was due to a negative relationship between self-investment and collective action when the negative emotions were highly salient, *β* = − .50, *t*(120) = 2.85, *p* = .005 (see Fig. 3). All other slopes were non-significant (*p*s > .10).

As can be seen in Fig. 3, people with high self-investment were less willing to engage in collective action when the negative emotions were salient than in any of the other conditions. Indeed, a comparison of the estimated means revealed that people with high (*M* + 1*SD*) self-investment were less likely to support collective action when negative emotions were salient (*M* = 2.14, *SE* = 0.31) than (1) when they were not (*M* = 3.49, *SE* = 0.39), *F*(1, 120) = 7.34, *p* = .008, *η*_p_^2^ = .06 and (2) when positive emotions were salient (*M* = 3.23, *SE* = 0.34), *F*(1, 120) = 5.82, *p* = .017, *η*_p_^2^ = .05.

We hypothesized that people would be less likely to support collective actions when positive emotions were salient. Simple effects analysis also revealed that people with low self-investment (*M* − 1*SD*) were less likely to support collective action when positive emotions were salient (*M* = 2.73, *SE* = 0.32) than when they were not (*M* = 3.77, *SE* = 0.31), *F*(1, 120) = 5.48, *p* = .021, *η*_p_^2^ = .04. Although people with high self-investment were also less likely to support collective action when positive emotions were salient (*M* = 3.23, *SE* = 0.34) than when they were not (*M* = 3.64, *SE* = 0.28), this difference was non-significant, *F*(1, 120) = 0.89, *p* = .347, *η*_p_^2^ = .01.

##### Negative anticipated group-based emotions

**Fig. 4:**
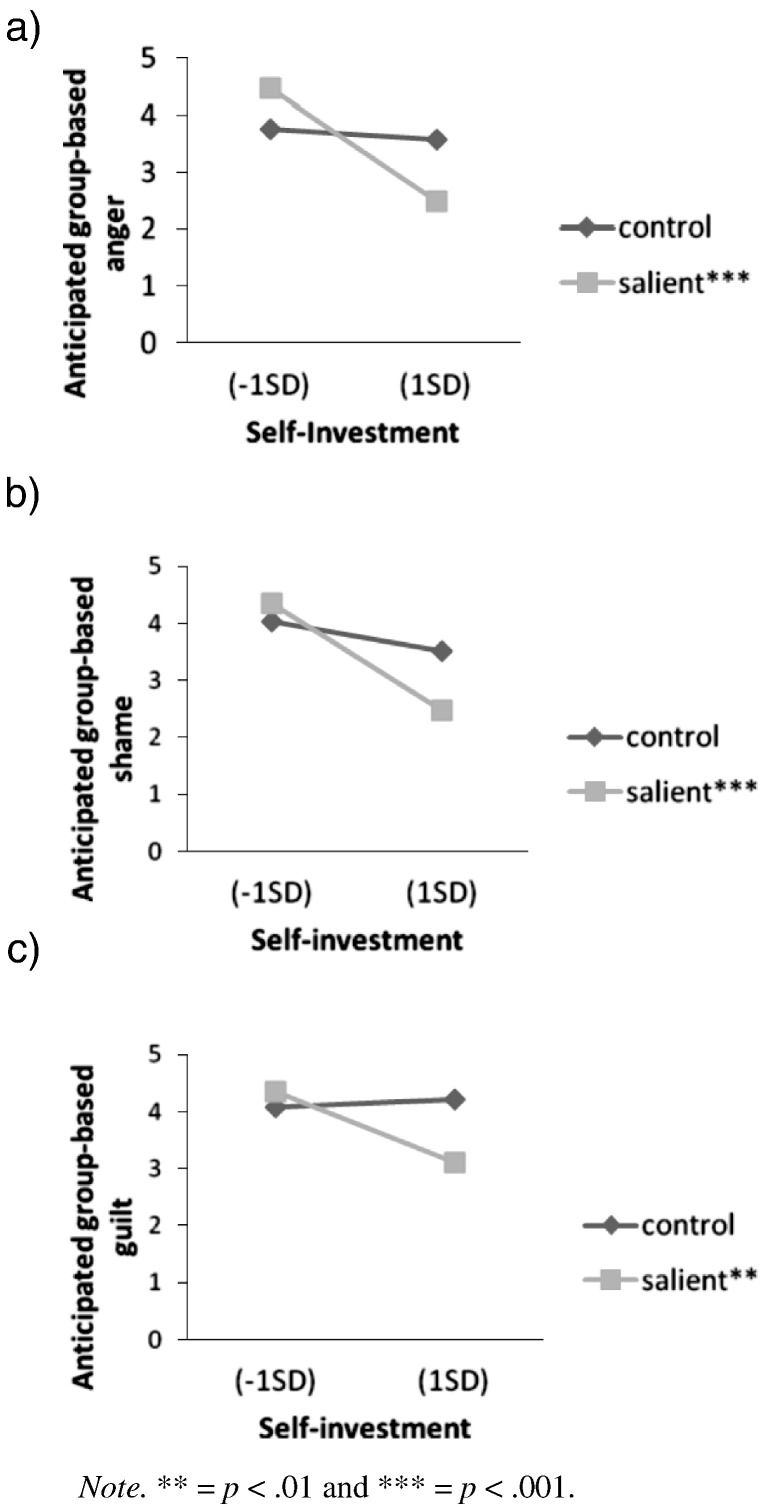
Interaction of salience and self-investment on anticipated group-based a) anger, b) shame, and c) guilt (Study 3). *Note*. **p < .01 and ***p < .001.

Anticipated group-based guilt, shame and anger were only rated in the negative valence condition. Participants in the positive valence conditions were therefore not included in these analyses and valence was not a factor. The main effect of salience on anger was not significant, *F*(1, 60) = 0.18, *p* = .674, *η*_p_^2^ < .01. Self-investment negatively predicted anger, *F*(1, 60) = 11.52, *p* = .001, *η*_p_^2^ = .16, (*β* = − .38, *p* = .001). This was qualified by a significant interaction between salience and self-investment, *F*(1, 60) = 7.69, *p* = .007, *η*_p_^2^ = .11. Simple slopes analysis revealed that self-investment negatively predicted anticipated group-based anger in the salient condition, *β* = − .69, *t*(60) = 4.67, *p* < .001, but not in the control condition, *β* = − .07, *t*(60) = 0.41, *p* = .680. As shown in Fig. 4a, people with low (*M* − 1*SD*) self-investment anticipated group-based anger to a greater extent in the salient condition (*M* = 4.48, *SE* = 0.31) than in the control condition (*M* = 3.71, *SE* = 0.28), *F*(1, 60) = 3.42, *p* = .069, *η*_p_^2^ = .05. Moreover, simple effects analysis revealed that people with high (*M* + 1*SD*) self-investment anticipated less group-based anger in the salient condition (*M* = 2.49, *SE* = 0.28) than in the control condition (*M* = 3.50, *SE* = 0.36), *F*(1, 60) = 5.09, *p* = .028, *η*_p_^2^ = .08.

Salience did not have a significant main effect on shame, *F*(1, 60) = 1.53, *p* = .222, *η*_p_^2^ = .03. Self-investment negatively predicted shame, *F*(1, 60) = 13.40, *p* = .001, *η*_p_^2^ = .18, (*β* = − .41, *p* = .001). This was qualified by a significant interaction between salience and self-investment, *F*(1, 60) = 4.40, *p* = .040, *η*_p_^2^ = .07. Simple slopes analysis revealed that self-investment negatively predicted shame in the salient condition, *β* = − .65, *t*(60) = 4.36, *p* < .001, but not the control condition, *β* = − .18, *t*(60) = 1.04, *p* = .303. As shown in Fig. 4b, people with high (but not low) self-investment anticipated group-based shame to a lesser extent in the salient condition (*M* = 2.48, *SE* = 0.28) than in the control condition (*M* = 3.52, *SE* = 0.36), *F*(1,60) = 5.28, *p* = .025, *η*_p_^2^ = .08.

The main effect of salience on guilt was non-significant, *F*(1, 60) = 1.87, *p* = .176, *η*_p_^2^ = .03. Self-investment did not significantly predict guilt, *F*(1, 60) = 2.47, *p* = .122, *η*_p_^2^ = .04. There was a near-significant interaction between salience and self-investment, *F*(1, 60) = 4.01, *p* = .050, *η*_p_^2^ = .06. Self-investment negatively predicted guilt in the salient condition, *β* = − .44, *t*(60) = 2.71, *p* = .009, but not the control condition, *β* = .05, *t*(60) = 0.29, *p* = .775. People with high (but not low) self-investment anticipated group-based guilt to a lesser extent in the salient condition (*M* = 3.11, *SE* = 0.30) than in the control condition (*M* = 4.22, *SE* = 0.38), *F*(1, 60) = 5.36, *p* = .024, *η*_p_^2^ = .08 (see Fig. 4c).

##### Positive anticipated group-based emotions

Neither the main effect of self-investment nor its interaction with salience had a significant effect on any of the positive anticipated group-based emotions (*p*s > .10).

#### Self-definition

The main effect of self-definition did not predict collective action, *F*(1, 120) = 1.35, *p* = .247, *η*_p_^2^ = .01. The interaction between salience and self-definition had a marginally significant effect on collective action, *F*(1, 120) = 2.98, *p* = .087, *η*_p_^2^ = .02.^7^ There was a marginally significant three-way interaction between salience, valence and self-definition, *F*(1, 120) = 2.82, *p* = .096, *η*_p_^2^ = .02. However, this interaction became non-significant when self-investment was entered into the model as a covariate, *F*(1, 119) = 2.66, *p* = .106, *η*_p_^2^ = .02. All other interaction effects on collective action were non-significant (*p*s > .10).

#### Reparation intentions

There were no significant main or interaction effects on reparation intentions (*p*s > .10). Because positive emotions were only rated in the positive valence condition and negative emotions were only rated in the negative condition, two analyses were conducted, one regressing reparation on positive anticipated emotions and the other regressing reparation on negative anticipated emotions. Across the two analyses, the only significant predictor of reparation intentions was anticipated group-based guilt, *β* = .49, *p* = .008.

#### Mediated moderation

We conducted mediated moderation analysis to determine whether the moderator (self-investment) affected the magnitude of the treatment effect (emotion salience) on the mediators (negative anticipated group-based emotions), which, in turn, predicted the outcome variable (collective action). Moreover, this analysis was also used to determine which negative anticipated group-based emotions were driving this effect. This mediating process could only be assessed for the participants in the negative valence condition because this is where negative emotions were measured. However, this is unproblematic because this is the condition in which a reduction in collective action was observed.

For participants in the negative valence condition, the effect of the salience by self-investment interaction on collective action intentions was marginally significant, *β* = − .21, *p* = .075, thereby fulfilling the first criterion for moderated mediation (Baron & Kenny, 1986; Preacher, Rucker, & Hayes, 2007). As reported above, the salience by self-investment interaction also affected the three negative anticipated group-based emotions, fulfilling the second criterion for moderated mediation. In keeping with Muller, Judd, and Yzerbyt (2005), the main effects of salience, self-investment, and anticipated group-based anger, shame, and guilt were entered into a regression equation. The interactions of self-investment with the salience manipulation and the three anticipated emotions were also entered into this regression equation.^8^ In this analysis, the salience by self-investment interaction was a non-significant predictor of collective action, *β* = − .05, *p* = .715. Anticipated group-based shame significantly predicted collective action, *β* = .48, *p* = .019. Anticipated group-based guilt and anger were non-significant predictors, *β* = − .11, *p* = .524 and *β* = .29, *p* = .132, respectively. All remaining predictors were non-significant (*p*s > .10). These results provided initial support for our mediated moderation hypothesis. Following Preacher et al. (2007), the significance of this indirect pathway was assessed using 95% bias-corrected confidence intervals, calculated using 5000 bootstrap resamples. This analysis assessed whether the effect of the salience by self-investment interaction on collective action was mediated by anticipated group-based shame. The confidence intervals did not include zero (CI_95_ = − .50, − .02), demonstrating a significant indirect effect. These results reflect the fact that the effect of the emotion salience by self-investment interaction on collective action is mediated by anticipated group-based shame.

### Discussion

In Study 3 we found that participants with high self-investment were less willing to engage in collective action in the negative emotion salience than in the control condition because they anticipated lower levels of group-based shame for undertaking the proposed transgression. Based on Studies 1 and 2, we hypothesized that this effect would also be mediated by anticipated anger. This was not the case, although it is worth noting that the effect of anger approached significance. We also hypothesized that people with low self-investment would exhibit greater levels of collective action when the negative emotions were salient than when they were not salient. In Study 3 this difference was non-significant. Previous research has found that low identifiers only undertake collective action when group efficacy is high (Van Zomeren et al., 2008). Because the participants were not informed about group efficacy, people with low self-investment may have been reluctant to undertake collective action when the negative emotions were salient.

In Study 3 we also investigated the effects of positive anticipated group-based emotions on collective action. We hypothesized that when positive anticipated group-based emotions were salient people would inhibit collective action, regardless of their level of self-investment. This hypothesis was supported for participants with low self-investment. These participants did show a reduction in collective action when the positive anticipated group-based emotions were salient, compared to the control condition, providing some support for our hypothesis. Although people with high self-investment were also less willing to undertake collective action in the positive emotion salient than the control condition, this difference was non-significant.

## General discussion

The aim of this research was to determine whether anticipating that an ingroup transgression would evoke aversive group-based emotions motivates group members to perform collective action to stop the transgression. In Study 1 we found that anticipated group-based shame and anger (but not guilt) positively predicted collective action against a proposed ingroup transgression and mediated the relationship between illegitimacy and collective action. Study 2 supported these results by finding that anticipated group-based shame and anger positively predicted collective action, and that shame (partially) and anger (fully) mediated the relationship between illegitimacy and collective action. Although Study 2 revealed a positive relationship between anticipated group-based guilt and collective action, further analysis revealed that guilt did not predict collective action taking either shame or anger into account (see Footnote 5). Moreover, in Studies 2 and 3 we found that anticipated group-based guilt predicted reparation for any negative consequences of the ingroup's actions. Previous research has found that group members sometimes use reparation to enhance social identity after experiencing group-based shame (Brown & Cehajic, 2008; Gausel, Leach, Vignoles, & Brown, 2012). In Study 3, the fact that anticipated group-based shame predicted collective action when another, less effortful affirmation strategy (reparation) was available implies that group members are motivated to undertake actions that prevent the identity-threatening situation rather than actions that may be used to repair one's identity at a later date.

In Study 3 we assessed whether participants high in self-investment would try to justify a future ingroup transgression when negative anticipated group-based emotions were salient, the aim of this justification being to protect the ingroup's identity. We found that participants with high self-investment exhibited reduced collective action intentions when negative anticipated group-based emotions were salient, compared to the control condition. This effect was mediated by anticipated group-based shame. This finding contrasts with the results of previous research in which it was found that people high in self-investment were most likely to exhibit *prosocial* behavior when they anticipated experiencing aversive group-based emotions for transgressions, in this case ingroup favoritism (Shepherd, Spears, & Manstead, 2012a). However, it should be noted that research has found that anticipated group-based shame is most likely to inhibit transgressions when the ingroup's status is high and secure (Shepherd, Spears, & Manstead, 2012b). In the present study, the threat posed by Iran's alleged nuclear weapons program may have motivated people with high self-investment to legitimize harmful actions in order to deal with the threat posed by the outgroup. This suggests that aversive anticipated group-based emotions are most likely to promote collective action against an impending transgression in less threatening circumstances.

We also hypothesized that the anticipation of positive group-based emotions would reduce participants' willingness to engage in collective action. In Study 3 we found that the anticipation of positive group-based emotions reduced collective action intentions in participants who were low in self-investment. Participants high in self-investment did exhibit a (non-significant) reduction in collective action when positive anticipated group-based emotions were salient.

Since the inception of intergroup emotion theory (Smith, 1993) there has been a growing interest in the motivational role of group-based emotions on collective action. Initially, this research focused on the role of group-based anger in motivating disadvantaged group members to perform collective action (Livingstone et al., 2009; Van Zomeren et al., 2004, 2008). Moreover, recent research has found that advantaged groups undertake collective action on the behalf of a disadvantaged group when they feel angry at the ingroup (Leach et al., 2006), sympathy towards the outgroup (Iyer & Ryan, 2009), or ashamed of their own group's actions (Iyer et al., 2007). To date, this literature has focused on the effect of directly experienced emotions on collective action. The present study extends this research by demonstrating that the anticipation of group-based emotions predicts people's willingness to undertake collective action against a proposed ingroup transgression. This research suggests that emotions do not need to be experienced in situ for them to affect the behavior of group members. The anticipation of an identity-threatening group-based emotion is sufficient to promote collective action.

As noted earlier, recent research has demonstrated that morality is a key aspect of ingroup identity (Ellemers et al., 2008; Leach et al., 2007; Pagliaro, Ellemers, & Barreto, 2011; Scheepers et al., 2009). Recently, Pagliaro et al. (2011) found that the effect of moral norms on group members' behavior was mediated by the anticipation of receiving respect from fellow group members, thereby suggesting that other-praising group-based emotions promote moral conduct. Although other-praising emotions are likely to influence moral conduct, a self-regulatory system is also required, otherwise “people would behave like weather vanes, constantly shifting direction to conform to whatever influence happened to impinge upon them” (Bandura, 2001, p. 7). Essentially, without a self-regulatory system people's behavior would be solely dependent on the perceived moral values of the group members that are present at a given time. We therefore extend the work of Pagliaro and colleagues by outlining a self-regulatory system for promoting moral intergroup behavior. In line with the interpersonal literature (Baumeister et al., 2007; Damasio, 1994; Haidt, 2001, 2007), we argue that anticipated group-based emotions help ingroup members to maintain a moral identity by signaling the aversive emotional consequences of proposed transgressions. This approach can be interpreted as a form of self- or ingroup-policing. The anticipation of these emotions warns ingroup members that the proposed actions of their group are immoral, which in turn motivates collective action to prevent the transgression and thereby maintain a moral group identity.

A growing body of research has investigated the role of positive group-based emotions in promoting non-egalitarian and aggressive ingroup actions. This research has found that group-based pride promotes ingroup bias (Harth et al., 2008), and that experiencing group-based satisfaction toward an aggressive ingroup action positively predicts support for future transgressions (Maitner et al., 2007). We extend this research by demonstrating that anticipating positive group-based emotions for a future ingroup transgression decreases the likelihood of ingroup members undertaking collective action against this behavior. This is not to say that positive group-based emotions always promote non-egalitarian and immoral intergroup behavior. Leach et al. (2007) found that group members experience pride when their group has a moral social identity. We believe that people may also anticipate group-based pride for egalitarian and prosocial behaviors, and that this may also promote moral intergroup actions. Further research is needed to investigate the effect of positive group-based emotions on moral intergroup behavior.

Haidt and colleagues (Graham et al., 2011; Haidt & Graham, 2007) suggest that five psychological systems provide the foundations of morality: harm/care, fairness/reciprocity, ingroup/loyalty, authority/respect, and purity/sanctity. In the present research we focused mainly on the role of anticipated group-based emotions in promoting egalitarian behavior (Study 1) and deterring harmful ingroup actions (Studies 2 and 3). This work may therefore be criticized for focusing primarily on the role of anticipated group-based emotions in promoting moral behavior relevant to the harm/care and fairness/reciprocity dimensions. However, we argue that these anticipated emotions are more relevant to fairness and care than to the other three dimensions. Group loyalty is an intra-group process and is therefore unlikely to be affected by anticipated *group-based* emotions. Similarly, although there may be an element of anticipated group-based guilt, anger and shame that promotes purity/sanctity, disgust is more characteristic in this domain (Cannon, Schnall, & White, 2011; Haidt & Joseph, 2007; Rozin, Lowery, Imada, & Haidt, 1999). Finally, in other research we have found that anticipated group-based guilt and shame did not affect a low-status group's discrimination against a high-status group (Shepherd et al., 2012b), suggesting that these emotions are unlikely to promote moral behavior in the authority/hierarchy domain. On the other hand, the fact that shame did down-regulate discrimination among those who already have high status suggests that it can foster more equal social relations.

Our results contrast with work by Tangney and colleagues (Dearing, Stuewig, & Tangney, 2005; Tangney & Dearing, 2002; Tangney et al., 2007) suggesting that moral behavior is more likely to be guided by guilt than shame. As noted earlier, recent research has found that shame can promote prosocial behavior (De Hooge et al., 2008, 2010), and that guilt has a ‘dark side’ (Bastian, Jetten, & Fasoli, 2011; De Hooge, Nelissen, Breugelmans, & Zeelenberg, 2011; Nelissen & Zeelenberg, 2009). One reason why Tangney and colleagues did not find a link between shame and prosocial behavior may be that they measured the participant's proneness to this emotion rather than the emotion itself (De Hooge et al., 2008) or its anticipated form as in the present research. In keeping with this idea, Tibbetts (1997) found that shame-proneness was positively associated with deviant behavior but that anticipated shame negatively predicted this action. Based on Tibbetts (1997) and recent research (e.g., De Hooge et al., 2008; Gausel & Leach, 2011), we argue that shame can promote moral behavior. The findings of the present studies extend this work by suggesting that (group-based) shame promotes moral intergroup behavior. It is also likely that because there is a greater distance between the individual and the transgression for both anticipated *and* group-based emotions, the identity threat posed by this action may be less harmful than if the individual had committed the transgression. This may make it less likely that people will implement defensive strategies in order to avoid shame.

There is some debate in the literature concerning the classification of anticipated emotions. Frijda (2004) suggested that anticipated emotions are predictive cognitions rather than emotions. However, there is evidence beyond our research that anticipated emotions elicit affective arousal. Imagery studies have found that imagining emotional experiences alters physiological arousal (e.g., Sinha, Lovallo, & Parsons, 1992). Similarly, participants exhibited higher skin conductance responses before undertaking a risky decision rather than a safe option (Bechara, Tranel, Damasio, & Damasio, 1996). In the latter study, the presence of this arousal suggests that a) anticipated emotions signal decisions that would be regretted, and b) these anticipated emotions are associated with affective reactions. The affective nature of anticipated emotions suggests that they are (cognitively-based) emotions rather than (or as well as) cognitions (Zeelenberg & Pieters, 2007).

It is worth considering the relationship between anticipated and directly experienced group-based emotions. Directly experienced emotions help to improve the accuracy of their anticipated counterpart (Baumeister et al., 2007; Damasio, 1994). People experience aversive moral emotions (such as guilt, shame, and self-directed anger) when their behavior violates a moral standard. The fact that the emotion is being experienced suggests that the anticipated emotions did not deter the immoral behavior and that the accuracy of this system needs to be improved. These directly experienced emotions become associated with the immoral behavior. The next time the person is in a situation in which this immoral action could be repeated, the anticipated emotion signals that this would result in aversive emotions. The anticipated emotion thereby helps to prevent the immoral action from being repeated. We believe that this process occurs at the intergroup level. Experiencing group-based guilt, shame or anger suggests that the ingroup has done something immoral. The aversive consequences of these directly experienced group-based emotions become associated with immoral action. When ingroup members find themselves in a position in which similar behavior could be enacted, anticipating these aversive group-based emotions should deter the immoral behavior: once bitten, twice shy. Put another way, those who do not learn from history are condemned to repeat it.

Turning to possible limitations of the present research, it could be argued that the observed effects are due to the anticipation of generalized negative affect, rather than specific emotions. Anticipating that the future actions of the ingroup would evoke negative arousal may have motivated ingroup members to undertake collective action against the proposed transgression. There are two reasons for rejecting this alternative explanation. First, in Study 2 the theoretical model in which the emotions were observed indicators of a latent negative affect variable did not fit our data well. This suggests that it was the anticipation of group-based emotions that predicted the action tendencies, rather than more generic negative affect. Second, if the results were caused by negative affect rather than the emotions, anticipated group-based shame, anger *and* guilt should predict collective action. The fact that the latter anticipated emotion consistently failed to uniquely predict collective action suggests that negative affect is not driving this process.

It could also be argued that, prior to a transgression, ingroup members may experience anger and shame at the hypocrisy of their leaders and that these *directly experienced* emotions promote collective action, rather than the anticipated emotions studied here. However we do not believe that this argument can account for our findings, for two reasons. First, although the salience of the anticipated emotions was manipulated between conditions in Study 3, the salience of directly experienced emotions was constant. The fact that we found a significant difference suggests that anticipated emotions are more likely to promote collective action, or explain the difference between conditions with respect to this outcome than the directly experienced emotions. Second, recent research suggests that directly experienced emotions can predict behavior indirectly through anticipated emotions (for a review, see Baumeister et al., 2007; Brown & McConnell, 2011). Indeed, a possibly legitimate criticism of the use of scenario studies in emotion research is that they assess anticipated emotions, rather than those directly experienced (although, as we argue, the net effect is likely to be similar). This suggests that group-based anger and shame may (at least partly) predict collective action through their anticipated counterparts. For example, feeling angry that members of one's ingroup are considering whether or not to perform a transgression may signal the aversive emotional consequences of performing this action (‘If I am angry that my group is considering this action; I am likely to be furious if they actually implemented this plan’). This anger may therefore predict collective action against a proposed ingroup transgression indirectly, through its anticipated counterpart. Moreover, as noted earlier, one reason why anticipated shame may be more predictive of preventive collective action than the directly experienced form of this emotion is that it may be less prone to the characteristic avoidant and distancing responses of shame (Tangney & Dearing, 2002; Tangney et al., 2007). Because something can still be done to prevent the shameful event from occurring, preventive rather than avoidant action tendencies may be promoted for the anticipated form. Future research could compare similar events that have taken place versus events that remain preventable to assess whether anticipated shame retains the positive behavioral correlates of shame while avoiding the negative ones.

In line with previous research (Iyer et al., 2007; Leach et al., 2006), we argued that anticipated group-based guilt is unlikely to predict collective action because the low action potential of guilt is insufficient to promote effortful behaviors. Moreover, anticipated group-based guilt is more likely to predict less effortful action tendencies, such as reparation. The results of the present studies supported these hypotheses. However, it should be noted that although research has investigated the low action potential of guilt at the interpersonal level (Frijda et al., 1989; Roseman et al., 1994), to our knowledge research has not assessed the action potential of *group-based* guilt relative to other emotions, such as group-based anger and shame. Intergroup emotion theory postulates that the key difference between interpersonal and intergroup emotions is the self (personal versus group-based) that provides a basis for appraising an event. Based on this rationale, group-based guilt should have a similar action potential to its interpersonal counterpart. However, further research is needed to substantiate this claim.

In the present research the collective action items assessed participants' willingness to undertake an action at the present time, whereas the reparation items asked participants to state their willingness to undertake an action at some point in the future. Anticipated group-based shame and anger predicted collective action, suggesting that these emotions promote immediate action that rectifies the situation. Anticipated group-based guilt positively predicted engaging in future reparation. Low action potential emotions (such as guilt) may be especially likely to predict actions that take place in the future because the temporal distance between the present and the future action reduces the amount of effort that needs to be expended right now. Moreover, group members may endorse such future actions in order to ‘cleanse their soul,’ thereby avoiding group-based guilt without actually doing anything.

In conclusion, the aim of this research was to determine whether the anticipation of aversive group-based emotions would motivate people to undertake collective action against a proposed ingroup transgression. Anticipated group-based shame and anger (but not guilt, see Footnote 5) predicted collective action in Studies 1 and 2. In Study 3, people with high self-investment were less willing to engage in collective action when negative anticipated group-based emotions were salient than they were in the control condition. This effect was found to be mediated by anticipated group-based shame. These studies extend the literature on collective action by showing that the anticipation of aversive group-based emotions can increase the likelihood of collective protest. This is important because previous research considering group-based emotions as predictors of collective action has focused on the presence of such emotions (e.g., anger) rather than their anticipated experience. Moreover, we extend recent developments in the interpersonal emotion literature by demonstrating that shame can serve to promote moral *intergroup* behavior.

## Figures and Tables

**Table 1:** Confirmatory factor analysis fit indices for the anticipated group-based emotions (Study 1).

	Fit indices							Model comparison with three-factor solution	
	*χ*^2^	df	GFI	CFI	NFI	RMSEA	AIC	∆*χ*²	df
Three-factor model	35.43†	24	.96	.99	.97	.053	77.43		
Two-factor combined guilt and shame model	48.74⁎⁎	26	.94	.98	.96	.072	86.74	13.31⁎⁎	2
Two-factor combined shame and anger model	114.95⁎⁎⁎	26	.83	.92	.90	.142	152.95	79.52⁎⁎⁎	2
Two-factor combined guilt and anger model	124.81⁎⁎⁎	26	.83	.91	.90	.150	162.81	89.38⁎⁎⁎	2
Single-factor model	141.71⁎⁎⁎	27	.80	.90	.88	.159	177.71	106.28⁎⁎⁎	3

*Note*. df = degrees of freedom; GFI = goodness-of-fit index; CFI = comparative fit index; NFI = normed fit index; RMSEA = root-mean-square error approximation; AIC = Akaike's information criterion.

† *p* < .10.

⁎⁎ *p* < .01.

⁎⁎⁎ *p* < .001.

**Table 2:** Descriptive statistics and inter-correlations between variables (Study 1).

	*M* (*SD*)	1	2	3	4	5
1. Illegitimacy appraisal	1.53 (0.25)	–				
2. Anticipated group-based guilt	2.68 (1.51)	.47⁎⁎⁎	–			
3. Anticipated group-based shame	3.24 (1.69)	.57⁎⁎⁎	.81⁎⁎⁎	–		
4. Anticipated group-based anger	2.94 (1.66)	.58⁎⁎⁎	.69⁎⁎⁎	.78⁎⁎⁎	–	
5. Collective action	2.98 (1.45)	.49⁎⁎⁎	.55⁎⁎⁎	.67⁎⁎⁎	.65⁎⁎⁎	–

*Note*. Table contains transformed illegitimacy appraisal variable.

⁎⁎⁎ *p* < .001.

**Table 3:** Descriptive statistics and inter-correlations between variables (Study 2).

	*M* (*SD*)	1	2	3	4	5	6
1. Illegitimacy	4.74 (1.98)	–					
2. Anticipated group-based guilt	2.97 (1.99)	.70⁎⁎⁎	–				
3. Anticipated group-based shame	3.31 (2.09)	.74⁎⁎⁎	.85⁎⁎⁎	–			
4. Anticipated group-based anger	3.15 (2.26)	.82⁎⁎⁎	.80⁎⁎⁎	.90⁎⁎⁎	–		
5. Collective action	3.79 (2.31)	.74⁎⁎⁎	.73⁎⁎⁎	.82⁎⁎⁎	.89⁎⁎⁎	–	
6. Reparations	1.44 (0.26)	.44⁎⁎⁎	.58⁎⁎⁎	.59⁎⁎⁎	.53⁎⁎⁎	.57⁎⁎⁎	–

*Note*. Table contains transformed reparation variable.

⁎⁎⁎ *p* < .001.

## Footnotes

1 The model fit was improved when the non-significant pathway between guilt and collective action was removed. We also measured group-based fear, anticipated group-based anger in the Welsh, anticipated group-based anger in significant others (Europeans), and the participant's belief that the Welsh would retaliate. Anticipated group-based shame and anger remained the strongest significant predictors of collective action when these variables were entered into the model. However, we did not include these variables in the final model in order to simplify the results.

2 By default, AMOS calculates the total significance of all indirect pathways. In order to assess each pathway separately, we constrained the relationship of illegitimacy to the anticipated emotions on the opposing pathways to zero. Further analysis suggested that there was no suppression in the model, making this an appropriate method to analyze these data.

3 We also attempted to manipulate illegitimacy by informing participants that it would be legitimate or illegitimate for Britain to use military force against Iran. However, this manipulation did not have a significant effect on any of the dependent variables (*p* > .10), and is therefore not discussed further.

4 Because we were unable to enter the three anticipated emotions into the same model, we were unable to determine the *unique* relationship of each anticipated emotion to collective action and reparation. The relationships described below are therefore not independent.

5 These paths should be interpreted with caution because the predictive power of each emotion is not independent. Indeed, the relationship between guilt and collective action became non-significant when either shame or anger was entered into this model (*β* = .07, *p* = .342 when controlling for shame, and *β* = .07, *p* = .210 when controlling for anger). Moreover, in these models both shame and anger predicted collective action (*β* = .54, *p* < .001 and *β* = .80, *p* < .001, respectively). The indirect pathway from illegitimacy to collective action via guilt also became non-significant when controlling for shame or anger (CI_95_ = − .01, .07, *p* = .218 and CI_95_ = − .01, .07, *p* = .193, respectively). Importantly, multicollinearity was not an issue in any of these analyses (lowest tolerance value = .23).

6 Further analysis revealed that this effect was due to the satisfaction and centrality subcomponents of self-investment, thereby supporting Leach et al. (2008).

7 Simple effects analysis revealed that people with high self-definition (*M* + 1*SD*) were less likely to engage in collective action when the emotions were salient (*M* = 2.60, *SE* = 0.25) than in the control condition (*M* = 3.74, *SE* = 0.24), *F*(1, 119) = 5.38, *p* = .022, *η*_p_^2^ = .04. We also found that the salience by self-definition interaction had a significant effect on anticipated group-based anger, *F*(1, 59) = 6.16, *p* = .016, *η*_p_^2^ = .10. People with high self-definition anticipated anger to a lesser extent in the salient condition (*M* = 3.30, *SE* = 0.28) than in the control condition (*M* = 4.02, *SE* = 0.31), *F*(1, 59) = 3.30, *p* = .074, *η*_p_^2^ = .05. Self-definition also had a significant main effect on guilt, *F*(1, 59) = 5.01, *p* = .029, *η*_p_^2^ = .08, reflecting a positive relationship between these variables (*β* = .32, *p* = .029).

8 The main effects and interactions of all the anticipated group-based emotions were entered into the regression equation to determine which emotion *uniquely* predicted collective action intentions. The salience by self-investment interaction still had a non-significant effect on collective action intentions when the anticipated group-based guilt and anger main effects and interactions were removed from the equation.
